# Molecular genetics of Chinese families with *TGFBI* corneal dystrophies

**Published:** 2011-02-04

**Authors:** Ting Zhang, Naihong Yan, Wenhan Yu, Yun Liu, Guo Liu, Xiaomei Wu, Jinxian Lian, Xuyang Liu

**Affiliations:** 1 Ophthalmic Laboratories & Department of Ophthalmology, West China Hospital, Sichuan University, Chengdu, P.R. China; 2 The Permanente Medical Group, Stockton, CA

## Abstract

**Purpose:**

To identify clinical features and mutations within the transforming growth factor-beta-induced (*TGFBI*) gene in three Chinese families with Granular corneal dystrophy, type 1 (GCD1) and Granular corneal dystrophy, type 2 (GCD2).

**Methods:**

Clinical features of GCD1 and GCD2 in three Chinese families were studied with slit-lamp and in vivo laser scanning confocal microscopy (LSCM). Molecular genetic analysis was performed on nine patients and fifteen unaffected individuals from these families. All exons of *TGFBI* were amplified by polymerase chain reaction (PCR) and sequenced.

**Results:**

Morphological changes in the cornea among affected individuals from three Chinese families examined by in vivo LSCM were almost the same. A heterozygous mutation C>T (R555W) was identified in exon 12 of *TGFBI* in patients of family A with GCD1. Another heterozygous mutation G>A (R124H) was found in exon 4 of *TGFBI* in affected members of family B and C with GCD2.

**Conclusions:**

Mutations R555W and R124H in *TGFBI* were identified in three Chinese families with GCD. Even though there are a variety of mutations in *TGFBI* of GCD, the different subtypes of GCD (GCD1, GCD2, and GCD3) are in fact the same disorder. Our work supports the hypothesis that corneal dystrophies with the common genetic basis in *TGFBI* should be grouped together as *TGFBI* corneal dystrophies.

## Introduction

Corneal dystrophies represent a group of inherited corneal diseases with progressive accumulation of deposits in different layers of cornea, resulting in loss of corneal transparency and visual impairment [1]. Identified genes responsible for corneal dystrophies include transforming growth factor-beta-induced (*TGFBI*), carbohydrate sulfotransferase 6 (*CHST6*), gelsolin (*GSN*), keratin 3 (*KRT3*), keratin 12 (*KRT12*), and chromosome 1, surface marker 1 (*M1S1*), with *TGFBI* mutations in human autosomal dominant corneal dystrophies being the most commonly observed.

*TGFBI* was first isolated in a study of genes induced by transforming growth factor-beta (TGFβ) from a human lung adenocarcinoma cell line in 1992 [2]. *TGFBI* encodes a 683 amino-acid protein containing an RGD motif (Arg-Gly-Asp) and four internal repeated domains which have highly conserved sequences founded in several species [3]. The most frequently reported sites of mutations are at positions 124 and 555 of TGFBIp [4], which were found to be within the fourth fasciclin-like domain [5]. TGFBIp, initially known as keratoepithelin (KE), is an extracellular matrix protein induced by TGFβ1. The role of *TGFBI* in the pathogenesis of the chromosome 5q31-linked corneal dystrophies still remain unclear, but TGFBIp is preferentially expressed on the extracellular surface of corneal epithelial cells [6] and pathologic deposits caused by TGFBIp accumulation were only observed in the cornea [7].

Sharing a common genetic origin, the hereditary corneal dystrophies linked to chromosome 5q31 and *TGFBI* include Reis-Bücklers corneal dystrophy (RBCD, also called Granular corneal dystrophy, type 3 [GCD3]), Thiel-Behnke corneal dystrophy (TBCD), Classic Lattice corneal dystrophy (LCD1), Granular corneal dystrophy, type 1 (GCD1) and type 2 (GCD2) [8,9]. GCD1, also called classic Granular corneal dystrophy, is characterized by discrete, rounded, crumb-like opacities in the anterior stroma of the central cornea. As the disease progresses, the lesions increase in size and number and may coalesce, extending anteriorly through breaks in Bowman’s membrane and posteriorly to the deeper stroma [3,10]. GCD2, originally known as Avellino corneal dystrophy, is featured by a combination of classic granular and lattice dystrophies. The corneal opacities are shaped like snowflakes, stars, or disks, together with linear opacities in typical cases [11]. Compared to GCD1, progression of GCD2 is slower and the visual acuity is less impaired [12].

In this study, we conducted a clinical evaluation and molecular genetic analysis in three Chinese families with GCD1 and GCD2 in southwest China.

## Methods

### Patients

**Figure 1:**
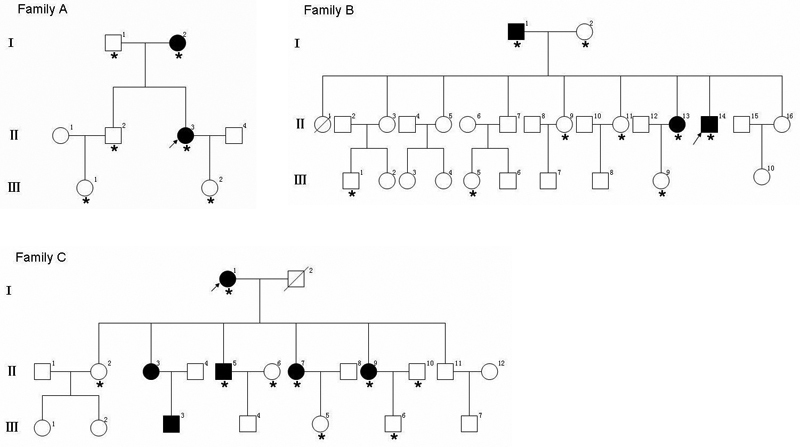
Pedigrees of three Chinese families. The pedigrees showed autosomal dominant inheritance of corneal dystrophies. The closed symbols represent subjects with GCD and the open symbols represent those who were not affected. Arrows indicate the probands. The asterisks indicate the individuals who had undergone clinical and molecular analyses in the study.

The study was approved by West China Hospital, Sichuan University Institute Review Board, Chengdu, Sichuan Province, P.R. China. Informed consent was obtained from all participants according to the tenets of Declaration of Helsinki. Nine affected and fifteen unaffected individuals, with ages ranging from 2 to 83 years old, from three unrelated Chinese families in Sichuan Province of Southwest China were enrolled in this study. No consanguineous marriage was noticed in these families (Figure 1).

### Clinical examination

All patients underwent ophthalmological examinations, including Snellen best-corrected visual acuity, detailed slit-lamp examination, and in vivo LSCM (Heidelberg Retina Tomograph III, Rostock Corneal Module [RCM]; Heidelberg Engineering GmbH, Heidelberg, Germany).

### Molecular genetic analysis

Peripheral blood was collected from each individual involved in this study. Genomic DNA was extracted from leukocytes using QIAamp DNA Blood Mini Kit (Qiagen, Hilden, Germany) by standard protocols. DNA integrity was evaluated by 1% agarose gel electrophoresis. Exons of *TGFBI* were amplified from genomic DNA of each participant by polymerase chain reaction (PCR) using the forward and reverse primers (Table 1). PCR was performed using 30 µl reaction mixtures, each containing 30–40 ng genomic DNA, 1.0 μM of each of the forward and reverse primers, and 15 μl of 2× Taq Master Mix (SinoBio Biltech Co. Ltd, Shanghai, China). Cycling conditions included an initial denaturation at 94 °C for 5 min, followed by 35 cycles of denaturation at 94 °C for 30 s, annealing at 52.6–64.3 °C for 30 s, extension at 72 °C for 30 s, and a final extension at 72 °C for 5 min. The amplified products were purified with a cycle-pure kit (OMEGA; Bio-Tek, Doraville, GA) and sequenced on the ABI 3730XL automated DNA sequencer (Applied Biosystems, Foster City, CA). Nucleotide sequences were compared with the wild type *TGFBI* sequence (GenBank NG_012646.1).

**Table 1:** Primers used in Polymerase Chain Reaction for amplification of *TGFBI*.

**Exon**	**Primer direction**	**Sequence (5′→3′)**	**Annealing temperature (°C)**	**Product length (bp)**
1	Forward	GCTTGCCCGTCGGTCGCTA	64.3	234
	Reverse	TCCGAGCCCCGACTACCTGA		
2	Forward	AGGCAAACACGATGGGAGTCA	60.1	204
	Reverse	TAGCACGCAGGTCCCAGACA		
3	Forward	CCAGATGACCTGTGAGGAACAGTGA	60.1	232
	Reverse	CCTTTTATGTGGGTACTCCTCTCT		
4	Forward	TCCTCGTCCTCTCCACCTGT	58.0	353
	Reverse	CTCCCATTCATCATGCCCAC		
5	Forward	CCTGGGCTCACGAGGGCTGAGAACAT	60.1	387
	Reverse	GCCCCTCTTGGGAGGCAATGTGTCCC		
6	Forward	CCTGGGCTCACGAGGGCTGAGAACAT	59.5	403
	Reverse	GCCCCTCTTGGGAGGCAATGTGTCCC		
7	Forward	GTGAGCTTGGGTTTGGCTTC	63.5	387
	Reverse	ACCTCATGGCAGGTGGTATG		
8	Forward	TGAGGTTATCGTGGAGTG	52.6	435
	Reverse	CACATCAGTCTGGTCACA		
9	Forward	ACTCACGAGATGACATTCCT	60.5	284
	Reverse	TCCAGGGACAATCTAACAGG		
10	Forward	TAGAAGATACCAGATGTTAAGG	56.1	426
	Reverse	TGTCAGCAACCAGTTCTCAT		
11	Forward	CCTGCTACATGCTCTGAACAA	58.0	321
	Reverse	GAATCCCCAAGGTAGAAGAAAG		
12	Forward	GACTCTACTATCCTCAGTGGTG	58.0	324
	Reverse	ATGTGCCAACTGTTTGCTGCT		
13	Forward	CATTAGACAGATTGTGGGTCA	59.9	419
	Reverse	GGGCTGCAACTTGAAGGTT		
14	Forward	GCGACAAGATTGAAACTCCAT	58.0	315
	Reverse	CTCTCCACCAACTGCCACAT		
15	Forward	CCCTCAGTCACGGTTGTT	58.0	348
	Reverse	GGAGTTGCCTTGGTTCTT		
16	Forward	CTTGCACAACTTATGTCTGC	58.0	319
	Reverse	TGCACCATGATGTTCTTATC		
17	Forward	AGTGAAGTTTCACAAACCAC	58.0	475
	Reverse	CCACATTTGGGATAGGTC		

Summary of the primers, annealing temperatures, and the predicted length for each of the PCR products is shown.

## Results

### Clinical findings

**Figure 2:**
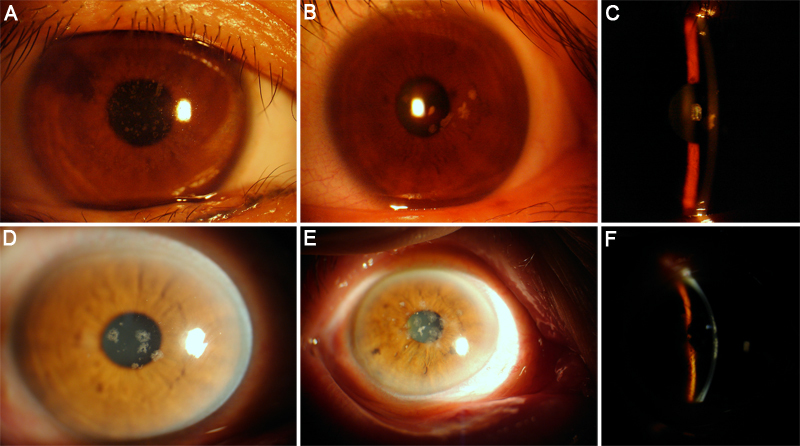
Slit-lamp photomicrographs. In family A, discrete dot opacities in the central cornea of the right eye were noted in the proband (**A**). In family B, the right eye of the proband showed a few grayish irregular and rounded corneal opacities (**B** and **C**). The peripheral cornea and the cornea between the opacities remained clear. In family C, the image of patient II:5 revealed scattered annular opacities (**D**). The proband presented with snowflake-like opacities combined with a linear opacity (**E** and **F**).

**Figure 3:**
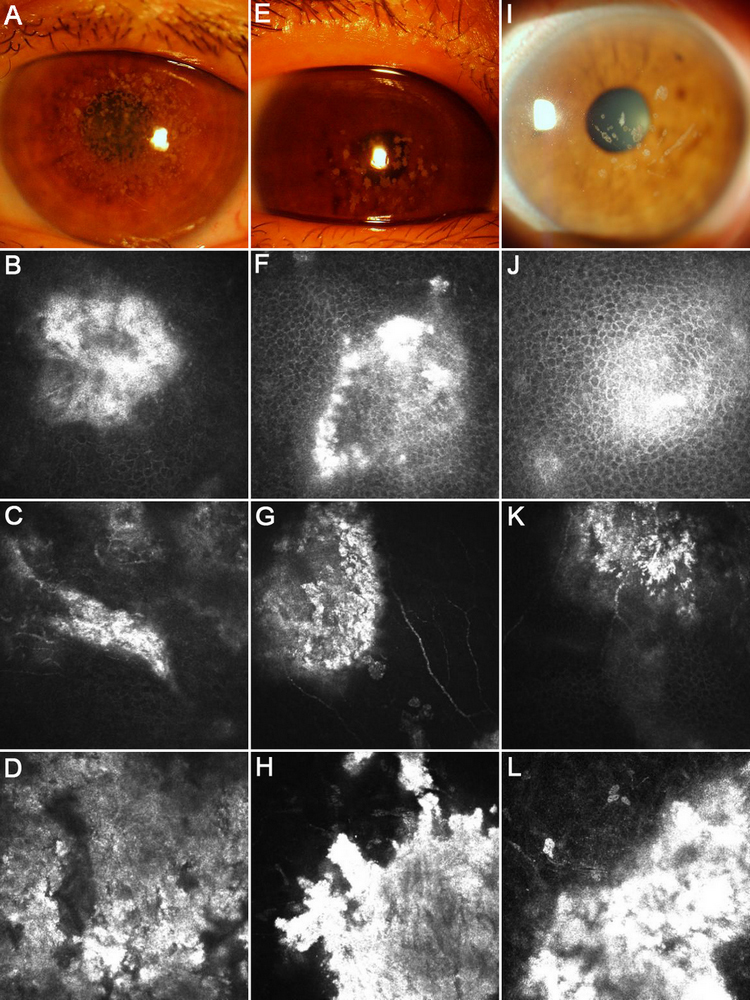
Representative images of affected members in the three families were compared by slit-lamp examination and in vivo LSCM. **A**-**D**: patient I:2 from family A; **E**-**H**: patient II:13 from family B; **I**-**L**: patient II:7 from family C. The eye of the proband’s mother revealed grayish annular and crumb-like opacities covered almost the entire cornea (**A**). The image of the proband’ sister showed multiple star-like and rounded opacities occupying the central cornea (**E**). The eye of the proband’s daughter presented with typical snowflake-like opacities combined with linear opacities (**I**). Focal deposits of reflective material with irregular edges were observed at different levels by LSCM. The images represented 23 μm (**B**, **F**, **J**), 45 μm (**C**, **G**, **K**), and 96 μm (**D**, **H**, **L**) from the corneal surface.

Nine individuals from the three families were diagnosed with GCD by clinical evaluation. The disease was bilateral in all patients. In family A, the proband (patient II:3, Figure 1) was a 26-year-old female presented with recurrent photophobia. The best corrected visual acuity was 20/20 OD and 20/20 OS. The corneal examination revealed discrete grayish dot-like opacities in the center of the cornea. The peripheral cornea and the cornea between the opacities remained clear (Figure 2A). The second patient from family A (patient I:2, Figure 1) was the proband’s mother (58 years old), who had decreased vision since the age of 40. Her best corrected visual acuity was 20/25 OD and 20/30 OS. Slit-lamp examination showed grayish annular and crumb-like opacities covered almost the entire cornea of both eyes (Figure 3A).

In family B, the proband, a 35-year-old male (patient II:14, Figure 1), was asymptomatic with a normal physical exam. He had a visual acuitiy of 20/20 OD and 20/20 OS. Slit-lamp examination revealed a few grayish irregular opacities located in the anterior corneal stroma (Figure 2B,C). Slit-lamp examination of another affected family member (the proband’s sister, patient II:13, Figure 1) showed multiple star-like and rounded opacities located in the anterior stroma of central cornea (Figure 3E).

In family C, the proband (patient I:1, Figure 1) was an 83-year-old female who first noticed blurry vision when she was 20 years old. She had experienced progressive deterioration in visual acuity bilaterally since then. Her current best corrected visual acuity was 20/200 OD and 20/100 OS. Slit-lamp examination revealed snowflake-like opacities combined with linear opacities in the anterior to middle stroma of the cornea in both eyes (Figure 2E,F). The proband’s daughter (patient II:7, Figure 1), who was 57 years old, exhibited more linear opacities in the cornea than the proband (Figure 3I). Patient II:5 (Figure 1), the 59-year-old son of the proband, manifested white annular opacities without linear changes (Figure 2D), which is quite different from the other patients in this family.

In vivo LSCM was performed on the subjects described above. Focal deposits of highly reflective material with irregular edges were observed in the corneal epithelium, anterior and middle stroma (Figure 3B-D,F-H,J-L).

### *TGFBI* gene analysis

**Figure 4:**
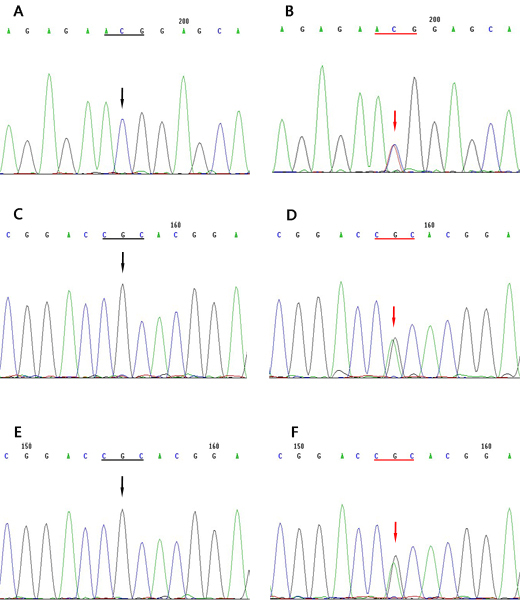
*TGFBI* heterozygous mutations in family A, B, and C. **A**: Unaffected individuals, no mutation was identified (black arrow). **B**: Patients in family A. A heterozygous mutation consisting of a C>T transversion in exon 12 (red arrow) was observed. **C** and **E**: Unaffected individuals, no mutation was detected (black arrow). **D** and **F**: The sequence in affected members in family B and C showed a heterozygous G>A transversion (indicated by the red arrow) was observed.

Seventeen exons of *TGFBI* of the affected and unaffected individuals were analyzed by direct sequencing. In family A, a heterozygous mutation C>T transversion in exon 12 was identified in each patient, which resulted in an amino acid substitution from Arginine (CGG) to Tryptophan (TGG) at codon 555 (R555W). In family B and C, a heterozygous mutation T>A in exon 4 was identified in each patient, which caused an amino acid substitution from Arginine (CGC) to Histidine (CAC) at codon 124 (R124H; Figure 4). No mutation was found in the unaffected members in these families.

## Discussion

Bilateral corneal dystrophy includes a group of genetically determined, non-inflammatory corneal diseases, which result in loss of corneal transparency and visual impairment [8,13]. In general, the dystrophies can be categorized according to the specific layer of cornea mainly affected; epithelial, subepithelial, Bowman’s layer, stromal, and endothelial dystrophies [14-17]. In this study, nine patients from three Chinese families presented with gray-white opacities in epithelial, subepithelial and anterior to middle stroma of both eyes. Thus, hereditary corneal dystrophies with atypical characteristics that affect multiple corneal layers can not be classified to a single type based on morphologic criteria [18]. The progress of molecular genetic analysis has deepened our understanding of the corneal dystrophies and showed the limitations of the current phenotypic method of corneal dystrophy classification. Since 1994 three types of autosomal dominant corneal dystrophies and *TGFBI* were mapped to chromosome 5 (5q31) [19,20], more than 30 mutations in *TGFBI* have been identified to be associated with corneal dystrophies with distinct subtypes being confirmed [17,19]. Mutations in varied genes can result in a single phenotype, whereas different defects in a single gene can cause various phenotypes. The IC3D Classification of Corneal Dystrophies, which incorporates many aspects of the conventional definitions of corneal dystrophies with new genetic, clinical, and pathologic information, provides a more accurate approach to classify corneal dystrophies including those with low likelyhood to have a genetic basis [8].

*TGFBI* is known to play a significant role in the pathogenic mechanism of most autosomal dominant corneal dystrophies including GCD1, GCD2, LCD1, RBCD, and TBCD. In our study, the affected members from family B and C were confirmed to share the same genetic mutation (R124H) in *TGFBI*. The patients from family B presented with atypical phenotype of GCD2, as their cornea had star-like and rounded opacities only (no linear opacities noticed), whereas the patients from family C manifested both typical and atypical phenotypes of GCD2. These observations indicate that GCD2 appears to be phenotypically variable and can be difficult to distinguish from GCD1 [12]. Clinical diagnosis of different types of corneal dystrophy is difficult, especially for GCD1 and GCD2. Molecular genetic analysis is required for subclassification of corneal dystrophies [21]. Identifying the causative gene is considerately significant to understand corneal disease not only for proper classification but also for prenatal diagnosis, accurate genetic counseling, or gene therapy [22].

LSCM, which is a non-invasive and real-time spatial sectioning of corneal tissues at the cellular level, was used in this study to better characterize corneal dystrophies and enhance our understanding of the clinical characteristics of different phenotypes of GCD. Previous study have shown that in GCD2, highly reflective granular materials with irregular edges were observed in the superficial stroma. In LCD1, highly reflective branching filaments of variable width were observed in the stroma. In macular corneal dystrophy (MCD), homogeneous reflective materials with dark striaelike images were observed throughout the stroma [23]. In our study, morphological studies of the GCD subjects from the three Chinese families under LSCM revealed focal deposits of reflective material with irregular edges at different layers of cornea. We previously reported the R124C mutation in *TGFBI* in RBCD (also known as GCD3) in a Chinese pedigree [24]. The results obtained from that pedigree and three pedigrees in this study suggested that even though clinical appearances under slit-lamp examination were different in these families, the morphological features of cornea deposits examined by LSCM were almost the same. For example, the irregular materials with high reflectivity in the epithelial basal layer in GCD3 [24] are similar to those observed in this study in GCD1 and GCD2 in both morphological features and laser light reflectivity. This illustrates that, even though there are a variety of mutations in *TGFBI* responsible for pathogenesis of GCD, the different subtypes (GCD1, GCD2, and GCD3) are in fact the same disorder.

Although the causative genes have been identified, the molecule mechanism and the pathogenesis of the diseases still remain to be illustrated. It was shown that mRNA of *TGFBI* is expressed in various organs and tissues [6]. The mutant TGFBIp deposits were only found in the cornea but not in other tissue of patients with corneal dystrophies [7], indicating that specific conditions existing in the cornea is responsible for the accumulation of mutated TGFBIp. Local factors may influence the TGFBIp amyloidogenesis [25]. Kim et al. [26] reported that periostin accumulated in deposits of aggregated mutant TGFBIp, suggesting that TGFBIp and periostin may play cooperative cellular roles in the pathogenesis of 5q31-linked corneal dystrophies. Furthermore, the study of mutated TGFBIp structure showed that the common mutations of *TGFBI* at mutation hotspots (positions 124 and 555) were likely to affect protein–protein interactions, whereas the rare mutations were inclined to cause misfolding of the protein within the cell [27]. In 1992, Korvatska [1] reported that two mechanisms of TGFBIp misfolding (amyloid and nonamyloid deposits) were implicated in the pathogenesis of the disease. The mutated TGFBIp comprised all kinds of pathological deposits; amyloid fibrils in LCD1 and non-amyloid amorphous accumulation in GCD1 and GCD3. GCD2, is a mixed form, containing both amyloid and nonamyloid aggregates [28]. The molecular basis underlying the formation of amyloid and/or nonamyloid accumulation is still unclear, and worthy of further studies.

In conclusion, this study elucidated the correlation between genotype and phenotype of GCDs. Our work supports the hypothesis that corneal dystrophies with the common genetic basis of *TGFBI* should be grouped together as *TGFBI* corneal dystrophies [8]. Obviously, more large pedigrees with GCD are needed in further investigations.
